# It's not fur: newspaper article reporting of abandonment and relinquishment of pets exhibit taxonomic biases in framing and language use

**DOI:** 10.1098/rsos.241958

**Published:** 2025-07-09

**Authors:** Jon Bielby, Kirsten McMillan, Georgie Davies

**Affiliations:** ^1^ School of Biological and Environmental Sciences, Liverpool John Moores University, Liverpool, UK; ^2^ Dogs Trust, London, UK; ^3^ Liverpool John Moores University, Liverpool, UK

**Keywords:** cats, dogs, exotic pets, rabbits, reptiles, sentiment analysis

## Abstract

Animal companionship holds cultural and economic significance, but there are downsides too, including welfare compromises, the risk of pathogen spillover and the relinquishment or abandonment of animals (cessation of ownership). The topic of cessation of ownership attracts a great deal of media coverage, and we hypothesize that biases exist in the amount and nature of coverage that different animal species receive, informing public views on its relative scale and importance across taxa. We searched English-language newspaper articles about cessation of ownership of pets to test whether major pet groups (cats and dogs; traditionally kept small mammals; reptiles and amphibians) differ in the number of newspaper article reports, their framing and their language use. Significant variation was observed, with a greater number of articles focused on cats and dogs. Reptile and amphibian articles were framed in a more abstract way, with more negative language. Newspaper articles write about reptiles and amphibians in a way that is consistent with ‘othering’, using words that exclude or dismiss another group. This trend may have important implications for public perceptions of the taxon, and any perceived lack of worth of a given taxon may feed into the support for interventions and policies aiming to optimize their welfare.

## 1. Introduction

Some of the largest proposed changes to animal-keeping legislation in the United Kingdom (UK) and European Union (EU) are focused on the distinction between so-called traditional companion animals and those considered to be non-traditional companion animals, sometimes known as ‘exotic’ pets (e.g. reptiles, non-poultry birds and non-domesticated mammals [1–5]). For example, at present in the UK and EU, exotic pets are the subject of research [3], advocacy campaigns [6] and policy position statements [7] in support of new legislation such as a ‘positive list’, under which only listed species are allowed to be kept as pets. Reptiles and amphibians are a commonly kept taxon that fall under the umbrella term ‘exotic pets’. A broad range of species of reptiles and amphibians are traded and kept as pets, many in large numbers. While it is extremely difficult to estimate the population of kept animals [8], an industry-led survey [9] annually reports the 10 most common pets within UK households. Dogs and cats are top ranking at 12 million (31% of households) and 11 million (26% of households), respectively, followed by rabbits at number 3 (1.5 million in 2.8% of households), indoor birds and domestic fowl at numbers 4 and 5 (1.3 million in 2.3% of households and 1.0 million in 1.3% of households, respectively), guinea pigs at number 6 (1 million in 1.8% of households), hamsters at number 7 (900 000 in 2.1% of households), tortoises and turtles at number 8 (900 000 in 1.8% of households), lizards at number 9 (800 000 in 1.6% of households) and snakes at number 10 (700 000 in 1.4% of households). These figures emphasize the size and diverse composition of the UK pet industry, reflecting its widespread appeal, and highlighting how common and popular reptiles are in the UK.

The large size of the pet industry and hobby includes a wide range of animals available within the market [10]. An unwanted corollary of the size and diversity of the trade is a considerable number of pet owners who are unable or unwilling to adequately provide for the physical, behavioural and environmental needs of their animal(s) [11–15]. This raises critical concerns regarding animal welfare and has sparked significant discourse concerning the implications of the pet trade, particularly within the exotics sector [6,15]. These animals may be relinquished or given up to sanctuaries or rescue centres [12,16–18], or abandoned, to either be discovered by others or fend for themselves out of a domestic setting [19,20]. For the purposes of this article, we attempt to use neutral language and make fewer assumptions about the motivations behind this phenomenon and so we use the umbrella term ‘cessation of ownership’ (or ‘cessation’ for brevity) rather than ‘rehome’, ‘relinquish’, ‘surrender’, ‘abandonment’ or ‘dump’, which vary in their meaning, perception and execution. As with overall population sizes of kept animals, it remains almost impossible to accurately quantify the number of ownership cessations and identify any temporal or taxonomic trends that exist. The difficulty in quantifying the number of cessations is largely due to the lack of legal definition of a rescue centre or sanctuary in most countries, and the associated lack of centralized, standardized record keeping [21]. Regardless of the overall quantity, the numbers are large, and rehoming, sanctuaries and/or rescue centres often run at full capacity [14]. Our perceptions of the size and severity of these challenges and our responses to them may not be proportionate across different taxa, as certain species groups typically receive more attention in the media and public eye than others [22–25]. Establishing whether this proportionality holds true is imperative if we want to promote responsible ownership practices within the pet-keeping community.

Public attitudes towards cessation in ownership and its perceived magnitude may be influenced by perceptions of the focal species, which may be shaped by their representation within media. Evidence suggests that human perception of reptiles and amphibians, or subclades within them (e.g. snakes), is generally negative. For example, according to a 2024 survey only 50% of the UK public think that snakes are sentient (i.e. can have physical and emotional experiences and can feel pain [26]), even though there is a growing body of evidence of advanced cognition [27–29] and reptiles’ ability to experience a range of emotions and states [30]. Further, in social media posts on the exotic pet trade, reptiles are significantly more likely to be the subject of negative comments [31], and comic book villains are more likely to have the names of reptiles or amphibians [32]. Meanwhile, in the non-virtual world, attitudes towards wildlife–vehicle collisions show significant taxonomic biases, with drivers being significantly less concerned with hitting snakes compared with other taxa [33]. Such taxonomic biases are important because human behaviours are guided by social values, public perception and attitudes, all of which can be driven by the media [34]. On an applied level, such media-driven perceptions could influence public opinions around the sentience, and expectations of the needs and husbandry requirements of a particular taxon. For example, recent research by a UK non-governmental organization suggests that prior to obtaining an exotic pet, many first-time owners have common misconceptions about their needs and the relative level of care that they require [15]. Thus, understanding the perceptions of different stakeholders in the sector (e.g. general public, first-time owners, traders and experienced keepers) and the sources of influence underpinning these perceptions may be crucial for the development and implementation of targeted interventions, aiming to reduce the frequency of cessation and for effective resource allocation proportional to the size of the issue in hand.

Communication style and language use construct our perception of reality and can be used to lead behaviour [35]. As such, the style and focus of media narratives and language used can make an applied link between our knowledge and the everyday, feeding into our choices and actions in the numerous roles we each fulfil in society (e.g. as consumers, pet-keepers, scientists, citizens, voters and policymakers [36]). The way in which the media communicates a subject of concern can, therefore, directly influence how people and society perceive, sometimes inaccurately, the urgency and magnitude of a given issue [37,38]. With this in mind, we may be interested in how communications on a given subject are ‘framed’, which is to say, which aspects of the situation or perceived reality are given more emphasis and made more salient, in a way that may lead to a particular evaluation, judgement or recommendation [34]. A common media frame paradigm is based on whether information is conveyed from the point of view of a specific case study or individual (episodic framing) or as a general theme or issue (thematic framing [39]; e.g. a case of a family living under economic duress versus general poverty rates increasing). Another commonly type is ‘valence framing’ [40], which focuses on the way in which information is presented to accentuate positive or negative aspects of a subject [41] (e.g. wins versus losses and survival versus mortality). Both of these types of framing in the media can influence people’s perceptions and decisions on a range of issues as diverse as poverty relief [39], sentencing for crimes [42] and the origin of manufacture of consumer products [43]. Consequently, acknowledging and accounting for framings and language use in media reporting is vital if we want public communications to be accurate in their portrayal of the size and severity of an issue (e.g. cessation of pet ownership) and therefore to develop ways to maximize the welfare of all pet animals.

Here, we examine how the topic of cessation of pet ownership is portrayed within English-language articles from UK newspapers, focusing on the influence of taxonomic group on the thematic versus episodic framing style used, and the language use and sentiment in these articles. Drawing on insights from prior research on human perceptions of animals [31–33], we hypothesize that articles addressing the cessation of ownership of reptiles and amphibians will be written in a more generic (thematic), less individual (episodic) style and will exhibit a more negative tone and language use compared with those discussing traditionally kept mammal species. Given the current scrutiny on the exotic pet trade within the UK’s devolved governments, we aimed to investigate whether different types of pets (taxonomic groups) lead to distinct framings in articles (episodic or thematic), and if there are variations in the emotion of the language used to describe cessation of pet ownership across taxonomic groups and framings.

## 2. Methods

### 2.1. ProQuest search terms

English-language articles related to the cessation of pet ownership were collected from UK newspapers in the ProQuest database. We selected ProQuest for our data collection because it centralizes sources from a broad range of other databases within which there are comprehensive collections of our media channel of choice (UK newspapers) over a suitable time span. We included articles from 2000 to 2023 inclusive and the search-terms used were ‘aban*’, ‘dump*’, ‘relinquish*’, ‘amphibian’, ‘bird’, ‘canary’, ‘cat’, ‘chicken’, ‘cockatiel’, ‘dog’, ‘ferret’, ‘finch’, ‘frog’, ‘guinea pig’, hamster’, ‘hedgehog’, ‘horse’, ‘lizard’, ‘newt’, ‘parrot’, ‘rabbit’, ‘reptile’, ‘salamander’, ‘snake’, ‘terrapin’, ‘toad’, ‘tortoise’ and ‘turtle’. The resulting articles were then filtered by eye independently by two authors (J.B. and G.D.) to remove duplicate articles and those that did not relate to the cessation of pet ownership via relinquishment or abandonment. We removed articles that focused on the following: the story behind the human or organization rather than the associated animals (e.g. focusing on the history of a person who had established a rescue centre); animals that were not kept as pets; feral populations rather than the cessation of ownership itself; dog theft; escaped animals; articles whose only content was touting for the adoption of specific individual animals; and pet or visitor attraction awards.

To quantify the framing and language use of each article, we decided to categorize whether articles were framed thematically (focusing on the general issue of cessation of ownership) or episodically (framed around an individual or group of individual’s story). For each article, we also wanted to quantify the tone of the language using a natural language processing approach implementing two separate lexicons. While language use and emotional language form an integral part of ‘valence framing’ [42], we refer to our sentiment analysis and language-use outputs simply as ‘language use’ rather than as a form of framing. We did so simply to avoid confusion with the analyses of thematic/episodic framing we present.

Each article was categorized according to the taxonomic group on which they focused, and whether the content was presented thematically or episodically (hereafter referred to as the ‘frames/framing’). Categorization of articles into episodic or thematic frames resulted in three categories that incorporated all articles while maintaining the overall purpose of our analyses. These were: (i) ‘general cessation issues’ (thematic category 1), focusing on large-scale ownership cessation and/or welfare concerns; (ii) ‘taxon-specific’ (thematic category 2), focusing on concerns for the welfare/cessation of ownership/illegal means of obtaining of a particular taxon; and (iii) ‘individual cessation’ (episodic category), focusing on a particular animal or group of animals that have been relinquished or abandoned. The latter category included dead animals but not animals that were assumed to have escaped but did not include articles on feral populations or animals in which it is possible that the animal(s) escaped. In cases where articles covered more than one of the above framing categories, they were recorded as ‘multiple’. After preliminary exploration of the way the articles were written and their content, we were able to categorize the articles into three exclusive taxonomic groups: ‘cats and dogs’, ‘traditional small mammals’ (consisting of rabbits, guinea pigs and other small traditionally kept rodents) and ‘reptiles and amphibians’. For example, an article could be assigned to the ‘reptiles and amphibians’ taxonomic category but still be placed in any of the three framing categories—a ‘general cessation issues’ article would focus on the issue in reptiles and amphibians generally without focusing specifically on one sub-group; a ‘taxon-specific’ article might cover cessation of ownership in a particular taxon within reptiles and amphibians (e.g. turtles); and an 'individual cessation’ framing would focus on the case and journey of a single animal (see [44] electronic supplementary material, File 1 for an example of each of these).

Articles were placed in the three framing categories outlined above by one author (G.D.), who did so based on reading the article title and main text and coded the framing directly into the dataset. To ensure repeatability regarding framing categorization, inter-rater reliability for assigning articles to specific categories was assessed using Cohen's kappa. A total of 200 papers were randomly selected and two of the authors (J.B. and G.D.) categorized them independently, and for each of the three framing categories, Cohen's kappa was calculated. The resulting metric ranges from 0 (complete disagreement) to 1 (perfect agreement). As a rule of thumb, Landis & Koch [45] suggested that Cohen's kappa of >0.8 = ‘almost perfect’, 0.61−0.79 = 'substantial', 0.41−0.59 = 'moderate', 0.21−0.39 = 'fair' and 0−0.2 = 'slight'. This method offers a more comprehensive measure than simple percentage agreement as it considers the likelihood of agreement being down to random chance.

The resulting dataset consisted of English-language newspapers articles from UK publications, and each of these had an associated framing and taxonomic focus (as defined above). This dataset allowed us to investigate whether the taxonomic focus of an article affects the framing used, and whether the language used to describe cessation of ownership or its effects varies between taxonomic groups and framing. All data collation, processing and statistical analyses were conducted in R [46] using the tidyverse package [47] and the dplyr library [48]. All data visualizations were made using ggplot2 [49].

### 2.2. Number of papers in each taxon and each category

To identify whether biases exist in how different taxonomic groups may be thematically or episodically framed, we counted the number of articles in each two-way combination of framing category and taxonomic focus, and the resulting contingency table was used as the basis of a Fisher’s Exact test. In the presence of any significant associations between framing category and taxonomic group, the standardized residuals of the contingency table were explored, and any with an absolute value of >2 were identified as being particularly important in driving the observed pattern [50].

### 2.3. Sentiment analysis of text

To investigate variation in language use between taxa and framing categories, two commonly used general purpose lexicons were implemented for sentiment analyses: *afinn* [51] and *nrc* [52]. Both lexicons contain many English-language words, and for a given passage of text, can be used to assign scores for positive/negative sentiment and specific emotions respectively. The *afinn* lexicon awards words a score between −5 and 5, with negative scores indicating negative sentiment and positive scores the opposite. To add further detail to our analyses, we also used the *nrc* lexicon, which categorizes words into two general sentiments (positive and negative) and eight specific emotions: anger, anticipation, disgust, fear, joy, sadness, surprise and trust. For each article, we first removed very common words that would be of little value in identifying differences in language use (e.g. ‘a’, ‘about’, ‘of’ and ‘the’). These are often called stop words [53], and in this case, we used those supplied in the R tidytext library [54]. We then converted the remaining text into tokens (in this case individual words). Tokenization aids more efficient data processing and allows sentiment analysis of the constituent words of a body of text. Finally, we stemmed all tokens to reduce them to their base or root form (e.g. ‘abandonment’ and ‘abandoned’ was reduced to ‘abandon’). We then calculated the net *afinn* score for each article, which was the sum total of the *afinn* scores of all of its constituent words. To use the finer scale *nrc* emotion categories, our metric of analysis was, for each article, the proportion of the total words in each of the eight *nrc* emotion categories. Text manipulation and cleaning was conducted using the stringr [55] and tidytext libraries [54] and word stemming used the wordStem function from the SnowballC library [56].

To compare the polarity of language in the articles written on different taxa, we used Kruskal–Wallis rank sum and then Dunn’s post hoc tests to compare the net *afinn* scores of articles written on dogs and cats, small traditional mammals, reptiles and amphibians. To determine whether our three taxa differed in the level of use of each of the eight *nrc* emotions in the language used, for each of the eight, we conducted a separate Kruskal–Wallis rank sum test followed by Dunn’s post hoc test to identify where differences between taxa existed, using with Bonferroni correction to adjust *p* values for multiple testing. For the purposes of sentiment analyses, we included only those articles that were exclusive to one taxonomic category. Further, because of the unbalanced and small sample sizes of intersections between taxon and framing type, we simply compared among taxa for all framings combined (i.e. the comparison was made among articles based on all those uniquely allocated to one of the three taxa regardless of whether the articles were framed episodically or thematically).

Because of the larger sample size within the articles focused on cats and dogs, for this taxon grouping, we compared the language use of different framing types (two thematic framing categories and one episodic framing category) using both *afinn* and *nrc* lexicons. To assess the relatively polarity of language use, we compared the net *afinn* scores of articles in each framing category using a Krukal–Wallis test and identified where differences lay using post hoc Dunn’s pairwise test. To compare the emotional content of each framing category, we compared those categories and the proportion of total words in each of the eight nrc emotions for each eight separately. Again, because of the non-normal nature of the data, we used Kruskal—Wallis tests with post hoc Dunn’s pairwise tests using Bonferroni correction to account for multiple testing.

## 3. Results

Data collection and quality control process yielded a total of 391 unique English-language newspaper articles published from 2000 to 2023 (included as electronic supplementary material, file 2 [57] in [25]). Cohen’s kappa suggests that these articles could be reliably categorized by independent observers for (i) general cessation of ownership articles (*n* = 24; Cohen’s kappa = 0.46 [0.60,0.73]; 87% agreement), (ii) taxonomically focused articles (*n* = 118; Cohen’s kappa = 0.59 [0.70,0.81]; 88% agreement) and (iii) individual articles (*n* = 121; Cohen’s kappa = 0.53 [0.64,0.74]; 83% agreement), and 128 articles contained more than one framing and were therefore categorized as having multiple frames. There were significant associations between the frequency of use of these categories and the taxonomic focus of the article (table 1; Fisher’s exact *p* < 0.001).

**Table 1.** Counts of article numbers with standardized residuals of observed and expected values in brackets for each combination of framing category and taxonomic focus. Negative standardized residuals mean there were fewer articles than expected by chance, positive standardized residuals mean there were more. Bold values were above the absolute value of 2.

	total	general abandonment (thematic frame 1)	taxonomic focus (thematic frame 2)	individual story (episodic frame)	multiple frames
**dogs and cats**	330	17 (−0.723)	104 (0.442)	116 (1.373)	93 (−1.446)
**traditional small mammals**	18	0 (−1.051)	8 (1.102)	1 (−1.936)	9 (1.280)
**herps**	43	**7 (2.684**)	6 (−1.937)	**4** (**−2.551**)	**26 (3.178**)

Exploration of the standardized residuals suggests that the significant associations between framing and taxon were heavily influenced by the underrepresentation of individual stories for reptiles and amphibians. Instead, reptiles and amphibians were more frequently discussed in a general context or with multiple framing elements than anticipated.

### 3.1. Sentiment analysis

**Figure 1.**
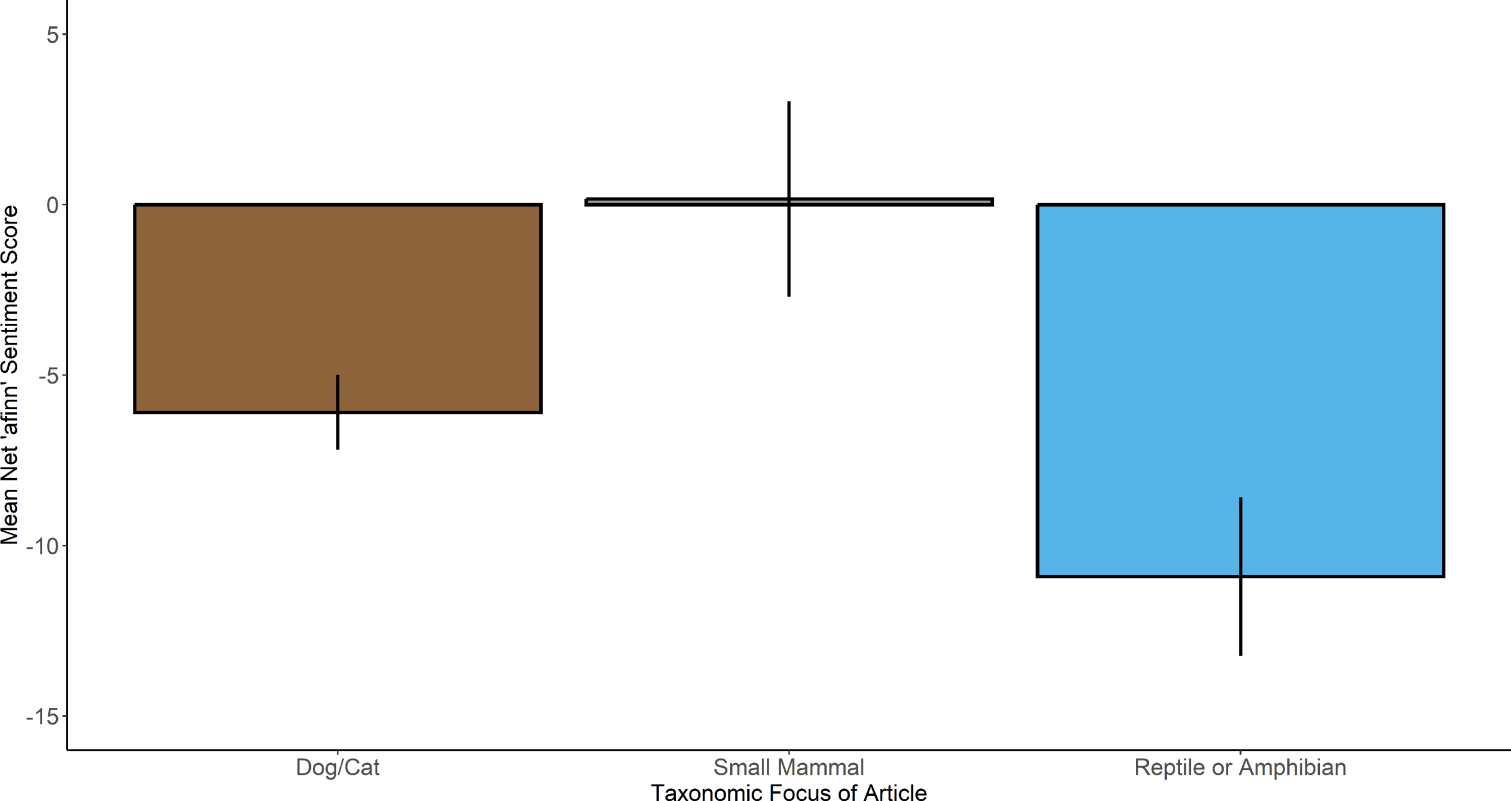
Comparison of mean (± s.e.) net affin scores of newspaper articles on the cessation of ownership of pets in different taxonomic categories. Articles on reptiles and amphibians used significantly more negative language than those written on traditional small mammals.

Analysis of net *afinn* sentiment scores among taxa suggest that significant differences occur among the groups (Kruskal–Wallis chi-squared = 6.72, d.f. = 2, *p* value = 0.03; figure 1). Post hoc tests identified that this difference was driven by articles on reptiles and amphibians being written in a significantly more negative way than those written on traditional small mammals (reptiles and amphibians, median = −9, interquartile range [IQR] = 14; traditional small mammals, median = 1.5, IQR = 11.75; *p* = 0.028). Neither differed significantly in net *affin* score from articles written on cats and dogs (median = −5, IQR = 21).

**Figure 2.**
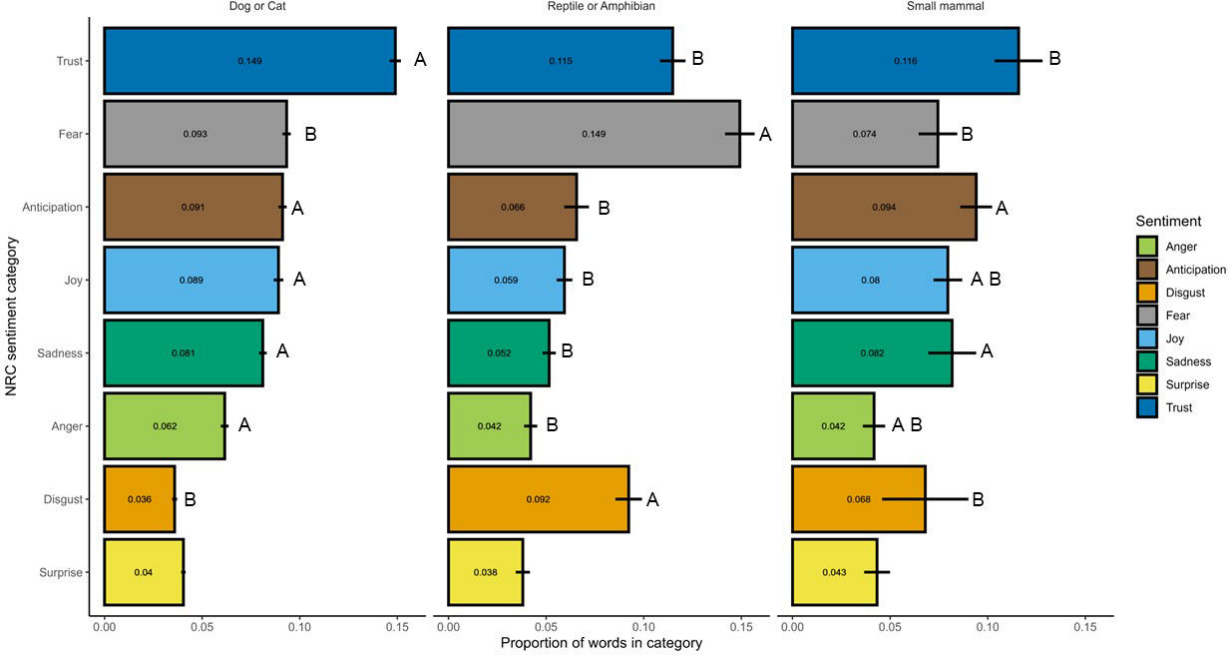
Mean (± s.e.) proportion of words per article in each nrc emotion category for each taxonomic group. Letters indicate groupings between which significant differences in proportion of those words exist based on Kruskal–Wallis test and Dunn’s pairs post hoc test (e.g. A and B significantly differ from each other, whereas AB does not differ from either A or B). Within an nrc emotion category, earlier letters indicate significantly higher mean proportion nrc scores within that taxon. The lack of a letter label indicates comparisons in which there was no significant difference between taxonomic categories.

Seven of the eight *nrc* emotion categories exhibited significant differences in their frequency of use between taxa (Kruskal–Wallis chi-squared = 21.7, d.f. = 2, *p* value < 0.001; figure 2), although the taxa between which the differences existed varied. The broadest overall pattern was that articles on reptiles and amphibians differed significantly in comparison to articles on dogs and cats in seven of the eight emotions and from small mammals in four of the eight emotions (figure 2). Reptiles and amphibians (R&A) were significantly higher than the other taxa in the amount of disgust (Kruskal–Wallis chi-squared = 58.88, d.f. = 2, *p* < 0.001; R&A (median and IQR) = 0.09 ± 0.07 versus cats and dogs [C&D] = 0.03 ± 0.03 and traditional small mammals [TSM] = 0.02 ± 0.08) and fear (Kruskal–Wallis chi-squared = 43.00, d.f. = 2, *p* < 0.001; R&A = 0.16 ± 0.09 versus C&D = 0.09 ± 0.05 and TSM = 0.07 ± 0.05). There were also significantly lower in anticipation (Kruskal–Wallis chi-squared = 19.67, d.f. = 2, *p* < 0.001; R&A = 0.06±0.03 versus C&D = 0.09 ± 0.05 and TSM = 0.09 ± 0.05), and sadness (Kruskal–Wallis chi-squared = 27.26, d.f. = 2, *p* < 0.001; R&A = 0.05 ± 0.03 versus C&D = 0.08 ± 0.05 and TSM = 0.08 ± 0.05). Cats and dogs were significantly higher than the other taxa in the use of language associated with trust (Kruskal–Wallis chi-squared = 19.87, d.f. = 2, *p* < 0.001; C&D = 0.14 ± 0.07 versus R&A = 0.10 ± 0.05 and TSM = 0.10 ± 0.05) and were significantly higher than reptiles and amphibians in language associated with joy (Kruskal–Wallis chi-squared = 14.92, d.f. = 2, *p* < 0.001; C&D = 0.08 ± 0.07 versus R&A = 0.05 ± 0.05; TSM = 0.08 ± 0.05), and anger (Kruskal–Wallis chi-squared = 15.80, d.f. = 2, *p* < 0.001; C&D = 0.06 ± 0.04 versus R&A = 0.04 ± 0.02; TSM = 0.04 ± 0.04). There was no difference in use of words associated with surprise among the taxa (Kruskal–Wallis chi-squared = 2.10, d.f. = 2, *p* = 0.35; C&D = 0.04 ± 0.03; R&A = 0.03 ± 0.03; TSM = 0.05 ± 0.04).

There were significant differences between polarity of language used in the different framing structures within the cats and dogs focused articles (Kruskal–Wallis chi-squared = 14.048, d.f. = 3, *p* value = 0.002). Episodically framed articles (i.e. those based around an individual animal; median = −1, iqr ± 23) were significantly less negative than those with a taxonomic focus (median = −16, iqr ± 19) or containing multiple framings (median = −11, iqr ± 24). Articles based around general cessation of ownership of cats and dogs did not differ from the other framing types in the net polarity of their language (median = −2, iqr ± 15).

**Figure 3.**
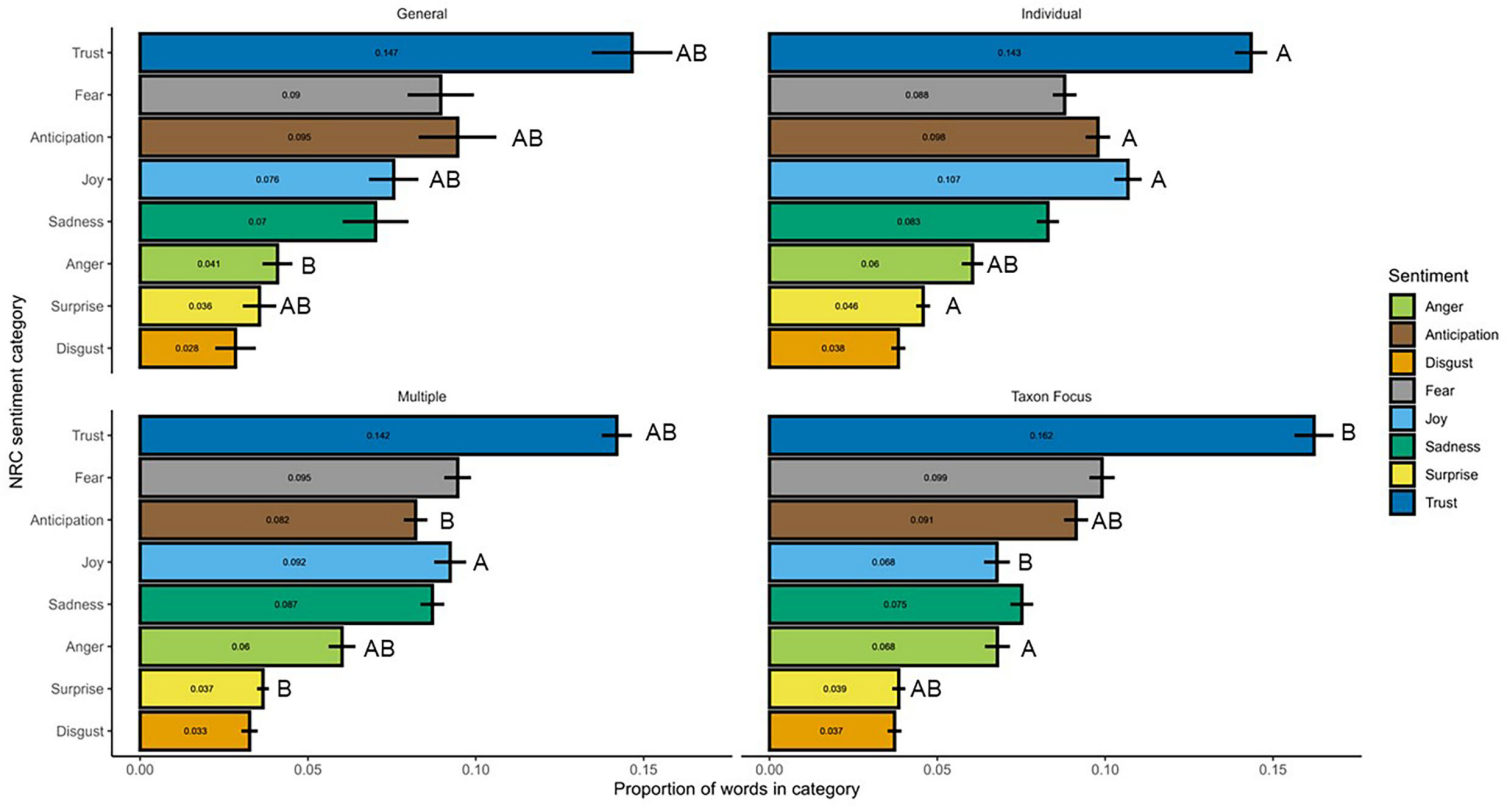
Mean (± s.e.) proportion of words per article in each nrc emotion category for each framing for articles based on cats or dogs. Letters indicate groupings between which significant differences in proportion of those words exist based on Kruskal–Wallis test and Dunn’s pairs post hoc test (e.g. A and B significantly differ from each other, whereas AB does not differ from either A nor B). Within an nrc emotion earlier letters indicate significantly higher mean proportion nrc scores within that taxon. The lack of a letter label indicates comparisons in which there was no significant difference between framing categories.

Comparison of nrc emotions across framing structures highlighted a clear division between language use in episodically framed articles compared with those with thematic frames. There was significant variation in language use among framing categories in five of the eight emotion categories, four of which involved a difference between the episodic frame and a thematic frame (figure 3). Words associated with disgust, fear and sadness showed no significant variation among framings.

## 4. Discussion

Keeping pets has a long history and can be culturally and economically important [58,59], but the practice comes with considerable drawbacks and costs, including the number of owners wanting or needing to relinquish, surrender, rehome or abandon their animals (cease their ownership) [12,16–20]. How society acknowledges, perceives and communicates this matter is important in shaping interventions aimed at reducing the issue. Where differences in societal perception and communication exist in reporting on taxonomic groups, there are likely to be challenges in developing and implementing interventions that are evidence-informed rather than being purely based on those perceptions. Our results suggest that while there are more articles on the cessation of ownership written about cats and dogs, which are the most kept UK pets, reptiles and amphibians are communicated about in a very different way, using very different language and emotions. Compared with both cats and dogs, and traditionally kept small mammals, cessation of ownership of reptiles and /or amphibians is typically discussed in a more general, less individual way, using more negative language associated with fear, disgust and with less joy and anticipation. So, while there are fewer articles on the subject in this taxon, articles that do exist are written about, and the taxon, therefore, likely perceived in a very different way than other taxa. It is, therefore, possible that these negative articles may feed into public perceptions and help shape opinions about the animals involved. In turn, these perceptions and opinions may influence and form the interventions that are developed with the aim of reducing the level of people wanting or needing to cease their ownership of these animals.

The large number of articles based around cessation of dog and cat ownership reflects the position of these species as the most popular pets within the UK [9]. While accurate population numbers are difficult to obtain, estimates suggest that there are over 12 million dogs [8] and over 10 million cats [60] within the UK. Correspondingly, in the UK, in keeping with other countries, the burden of cats and dogs being relinquished, surrendered or abandoned is considerable [61–64]. It is, therefore, no surprise that these widely kept species with complex needs are most frequently covered in newspaper articles regarding the subject. What is perhaps surprising is the relatively low number of articles on small traditionally kept mammals, reptiles and amphibians and the contrasting language use in articles on the three taxa. While dogs and cats dominate the number of individual animals kept the number of newspaper articles on ownership cessation does not directly reflect the population size. The best resource we have for comparing the relative popularity of pet types in the UK is the UK Pet Food annual survey (formerly Pet Food Manufacturers' Association) [9], and based on its estimates, the relative paucity of articles on traditional small mammals is striking—using our count of articles and the Pet Food Manufacturers' Association population data, there is approximately 1 article per 30 000 reptiles, per 72 000 dogs and cats and per 128 000 traditional small mammals. This latter group has a much lower per capita rate of articles on the subject compared with the other taxa, and while possible, it seems unlikely that this purely reflects a lower rate of people needing or wanting to cease ownership of their animals. The exact number of animals being relinquished or abandoned and reason(s) behind it is hard to quantify—but the level is not insignificant [12,18], and there are considerable welfare challenges and knowledge gaps related to the husbandry of rabbits, guinea pigs and small rodents [65]. Our analyses suggest that both the low number of newspaper articles on ownership cessation, and the generally positive language used in those articles, may undersell the size of the issue, thus diminishing the profile and severity of welfare concerns within the rabbit, guinea pig and hamster trade.

In contrast, reptiles and amphibians have the most articles on relinquishment and abandonment per capita of the estimated pet population, which exhibit significant differences in the framings and language use between taxa. Whereas cat- and dog-focused articles were often framed episodically, being based around an individual animal’s story, this is much less often the case in relative terms for reptiles and amphibians and for small mammals. The focus on an individual framing for cats and dogs likely reflects the tendency of society and the popular media to perceive dogs and cats as animals with which they can develop strong human–animal bonds, and perhaps even consider a member of the family [66,67]. Conversely, reptiles- and amphibian-focused articles commonly featured a framing based around the general issue of ‘exotic’ pets or reptiles rather than containing an individual’s story, which is consistent with them being objectified rather than being perceived as individual beings. In fact, regardless of society’s views on the suitability of reptiles and amphibians as pets, there is growing evidence that reptile keepers form strong bonds with their animals [4,68], and that reptiles and amphibians are capable of advanced cognitive functioning [29,69,70] and even self-recognition [71], a level of complexity and sentience belied by newspaper coverage and language use.

There are justifiable concerns about the suitability of reptiles as pets [6], and whether reptile keepers have sufficient information to adequately care for their animals [68,72]. However, reptile owners tend to have a higher awareness of their cognitive abilities and welfare needs than non-owners [73], suggesting that familiarity with and exposure to reptiles can help manage the expectations and perhaps change behaviour and perceptions around the taxon. The contrast between how reptiles and amphibians is written about in newspaper articles (i.e. thematically framed, using negative language with high levels of fear and disgust) and their complex captive needs and advanced cognitive abilities is striking. This disjunct should be acknowledged by communicating and highlighting the sentience, advanced cognitive capabilities and welfare needs of reptiles in ways that foster sustainable behaviours in the sector. It is important to effectively understand the motivations of and perceptions around reptile and amphibian ownership and drivers of its cessation and to develop interventions aimed at reducing their frequency. However, it is unclear how the broad, abstract framing and use of strong negative emotional language around the subject of reptile relinquishment or abandonment, which is typical of ‘othering’ [74], allows room to foster the behaviour changes necessary to resolve the issue.

The scale of our analysis does not allow us to identify the drivers of more generic, thematic framing and negative language in reptiles and amphibians. It is possible that the reasons are related to poor perceptions of the animals themselves, the practice of keeping them and/or their relinquishment/abandonment and its nature. The latter two could encourage readers to think more carefully before buying a reptile/amphibian thereby reducing the risk of this end point. Further, the framing choices, language use and sentiments expressed could be related to the experiences and preferences of the editors, journalists, newspapers and their readerships of the articles in question. Content analysis of popular media and interviews with people who have abandoned or relinquished their animals, and with journalists covering such stories could be an interesting way to examine the relationship between media communications and the taxon. Details notwithstanding, the lack of positive language (measured via afinn) and largely negative emotional words used (measured via ncr), along with other subconscious biases against reptiles and amphibians [31–33] suggests that our relationship with the animals themselves tends to be different to the one we hold with more traditional, mammalian pets.

Future research could explore various avenues with regards to media biases within the pet trade and their influence. While news media, whether hard copy or online, can still influence public and industry [75], other forms of media may also play a similar role in society [76,77]. Social media, for example, can illustrate and perhaps even drive discrepancies between public perception of an issue and the weight of scientific evidence on that matter [37,38]. Furthermore, links have been made between social media influencers and positive public attitudes towards exotic pet keeping and trade [31]. A better understanding of the causal links between social media output, attitudes towards and welfare needs of any group of animals is a key in developing more ethical and sustainable behaviours within the UK trade, the pet-keeping hobby and the general public. Here, we focused on the UK because the subject of exotic pets is under a great deal of scrutiny and political discussion [5], and the perceived high levels of abandonment and relinquishment are important drivers of this increased attention. However, we recommend that future researchers conduct comparative studies in other countries to explore varying attitudes towards pet ownership alongside varying historical, cultural and societal norms, which may lead to different attitudes towards pet ownership [2,78–80] and animal welfare more generally [81,82].

The UK pet trade is large and has a considerable economic worth [9]. Associated with the magnitude of the hobby and trade are several high-profile challenges [12,83,84], some of which are related to welfare concerns about the exotic pet trade/hobby [6,10], specifically that focused on reptiles and amphibians [72]. However, given the diversity of reptile and amphibian species traded and kept [85], we lack quantitative data on trade dynamics [86], detailed knowledge of their welfare needs [87,88] and suitable metrics for measuring their welfare [89]. These challenges have led to calls and communications on tightening regulations around the trade/hobby of keeping reptiles and amphibians [5,7]. The results presented here suggest that in relation to cessation of pet ownership, reptiles and amphibians are presented as a collective rather than as individuals and are discussed in a negative manner. It is unknown how this framing and language use translate to public and societal attitudes and actions, but we need to acknowledge and account for these biases when developing interventions if we want them to be evidence-informed and effective rather than driven by the emotion of communications.

## Data Availability

The dataset used for all analyses is available on Dryad [44].

## References

[1] Carpenter AI, Andreone F, Moore RD, Griffiths RA. 2014 A review of the international trade in amphibians: the types, levels and dynamics of trade in CITES-listed species. Oryx **48**, 565–574. (10.1017/s0030605312001627)

[2] Alves RRN, de Araújo BMC, da Silva Policarpo I, Pereira HM, Borges AKM, da Silva Vieira WL, Vasconcellos A. 2019 Keeping reptiles as pets in Brazil: ethnozoological and conservation aspects. J. Nat. Conserv. **49**, 9–21. (10.1016/j.jnc.2019.02.002)

[3] Toland E, Bando M, Hamers M, Cadenas V, Laidlaw R, Martínez-Silvestre A, van der Wielen P. 2020 Turning negatives into positives for pet trading and keeping: a review of positive lists. Animals **10**, 2371. (10.3390/ani10122371) 33322002 PMC7763047

[4] De la Fuente MF, de Araújo BMC, da Silva Policarpo I, Pereira HM, Borges AKM, Vieira WLS, Pereira Filho GA, Alves RRN. 2023 Keeping reptiles as pets in Brazil: keepers’ motivations and husbandry practices. J. Ethnobiol. Ethnomedicine **19**, 46. (10.1186/s13002-023-00618-z) PMC10590521 37865770

[5] Scottish Animal Welfare Commission. 2022 Exotic pet working group—final report. Scottish Government. See https://www.gov.scot/publications/final-report-exotic-pet-working-group-scottishanimal-welfare-commission/ (accessed May 2024).

[6] Born Free Foundation and the Royal Society for the Prevention of Cruelty to Animals. 2021 The exotic petdemic: UK’s ticking timebomb exposed. See https://www.rspca.org.uk/documents/1494939/7712578/The+Exotic+Pet-demic%3A+UK%27s+ticking+timebomb+exposed.pdf/075754a7-fa68-f9bf-66b4-ccb0d559db28?t=1631617196174 (accessed 1 May 2024).

[7] British Veterinary Association. 2023 Policy position on non-traditional companion animals. See https://www.bva.co.uk/take-action/our-policies/exotic-pets-non-traditionalcompanion-animals/ (accessed 1 May 2024).

[8] McMillan KM, Harrison XA, Wong DC, Upjohn MM, Christley RM, Casey RA. 2024 Estimation of the size, density, and demographic distribution of the UK pet dog population in 2019. Sci. Rep. **14**, 31746. (10.1038/s41598-024-82358-y) 39738471 PMC11686149

[9] Pet Food Manufacturers Association. 2023 UK pet food. See https://www.ukpetfood.org/information-centre/statistics/uk-pet-population.html (accessed 1 May 2024).

[10] Elwin A, Green J, D’Cruze N. 2020 On the record: an analysis of exotic pet licences in the UK. Animals **10**, 2373. (10.3390/ani10122373) 33322026 PMC7763562

[11] Casey RA, Vandenbussche S, Bradshaw JWS, Roberts MA. 2009 Reasons for relinquishment and return of domestic cats (Felis silvestris catus) to rescue shelters in the UK. Anthrozoös **22**, 347–358. (10.2752/089279309x12538695316185)

[12] Ellis CF, McCormick W, Tinarwo A. 2017 Analysis of factors relating to companion rabbits relinquished to two United Kingdom rehoming centers. J. Appl. Anim. Welf. Sci. **20**, 230–239. (10.1080/10888705.2017.1303381) 28429961

[13] Fernandez EB, De Blas Giral I, Thiemann AK, Vázquez Bringas FJ. 2021 Demography, preventative healthcare and reason for relinquishment of donkeys to an equine charity in the UK (2013‐2015). Equine Vet. J. **53**, 324–330. (10.1111/evj.13310) 32542888

[14] Powdrill-Wells N, Taylor S, Melfi V. 2021 Reducing dog relinquishment to rescue centres due to behaviour problems: identifying cases to target with an advice intervention at the point of relinquishment request. Animals **11**, 2766. (10.3390/ani11102766) 34679789 PMC8532592

[15] RSPCA. 2017 Understanding the motivations of beginner reptile owners. Horsham, UK: Royal Society for the Prevention of Cruelty to Animals. See https://www.rspca.org.uk/webContent/staticImages/Downloads/ReptileReport.pdf (accessed 1 May 2024).

[16] Clark CC, Gruffydd‐Jones T, Murray JK. 2012 Number of cats and dogs in UK welfare organisations. Vet. Rec. **170**, 493–493. (10.1136/vr.100524) 22589036

[17] Coe JB, Young I, Lambert K, Dysart L, Nogueira Borden L, Rajić A. 2014 A scoping review of published research on the relinquishment of companion animals. J. Appl. Anim. Welf. Sci. **17**, 253–273. (10.1080/10888705.2014.899910) 24738944

[18] Neville V, Hinde K, Line E, Todd R, Saunders RA. 2019 Rabbit relinquishment through online classified advertisements in the United Kingdom: when, why, and how many? J. Appl. Anim. Welf. Sci. **22**, 105–115. (10.1080/10888705.2018.1438287) 29508633

[19] Stringham OC, Lockwood JL. 2018 Pet problems: biological and economic factors that influence the release of alien reptiles and amphibians by pet owners. J. Appl. Ecol. **55**, 2632–2640. (10.1111/1365-2664.13237)

[20] Bernete Perdomo E, Araña Padilla JE, Dewitte S. 2021 Amelioration of pet overpopulation and abandonment using control of breeding and sale, and compulsory owner liability insurance. Animals **11**, 524. (10.3390/ani11020524) 33670459 PMC7922531

[21] Dogs Trust. 2024 Our manifesto: a better future for dogs. See https://www.dogstrust.org.uk/support-us/campaigns-appeals/manifesto (accessed 12 March 2025).

[22] Troudet J, Grandcolas P, Blin A, Vignes-Lebbe R, Legendre F. 2017 Taxonomic bias in biodiversity data and societal preferences. Sci. Rep. **7**, 9132. (10.1038/s41598-017-09084-6) 28831097 PMC5567328

[23] Hinsley A, Hughes A, Margulies J. 2024 Creating a more inclusive approach to wildlife trade management. Conserv. Biol. **38**, e14360. (10.1111/cobi.14360) 39248773

[24] Hu S, Liang Z, Liang D, Liu Y, Zhong J, Wei Q, Lee T. 2024 Quantifying species biases among multidata sources on illegal wildlife trade and its implications for conservation. Conserv. Biol. **38**, e14351. (10.1111/cobi.14351) 39248759

[25] Bielby J, Austen G, McMillan K, Wafflart S. 2025 Exploring media representation of the exotic pet trade, with a focus on welfare: taxonomic, framing and language biases in peer-reviewed publications and newspaper articles. R. Soc. Open Sci. **12**, 240952. (10.1098/rsos.240952) 40046661 PMC11880843

[26] RSPCA. 2024 Kindness index. See https://www.rspca.org.uk/whatwedo/latest/kindessindex/annual/report2024#top (accessed 7 August 2024).

[27] Wilkinson A, Kuenstner K, Mueller J, Huber L. 2010 Social learning in a non-social reptile (Geochelone carbonaria). Biol. Lett. **6**, 614–616. (10.1098/rsbl.2010.0092) 20356886 PMC2936136

[28] Kis A, Huber L, Wilkinson A. 2015 Social learning by imitation in a reptile (Pogona vitticeps). Anim. Cogn. **18**, 325–331. (10.1007/s10071-014-0803-7) 25199480

[29] Matsubara S, Deeming DC, Wilkinson A. 2017 Cold-blooded cognition: new directions in reptile cognition. Curr. Opin. Behav. Sci. **16**, 126–130. (10.1016/j.cobeha.2017.06.006)

[30] Lambert H, Carder G, D’Cruze N. 2019 Given the cold shoulder: a review of the scientific literature for evidence of reptile sentience. Animals **9**, 821. (10.3390/ani9100821) 31627409 PMC6827095

[31] Anagnostou M, Doberstein B. 2024 Exotic pet trade in Canada: the influence of social media on public sentiment and behaviour. J. Nat. Conserv. **77**, 126522. (10.1016/j.jnc.2023.126522)

[32] Geest EA, Knoch AR, Shufran AA. 2022 Villainous snakes and heroic butterflies, the moral alignment of animal-themed characters in American superhero comic books. J. Graph. Nov. Comics **13**, 735–750. (10.1080/21504857.2021.1998173)

[33] Crawford BA, Andrews KM. 2016 Drivers’ attitudes toward wildlife‐vehicle collisions with reptiles and other taxa. Anim. Conserv. **19**, 444–450. (10.1111/acv.12261)

[34] Entman RM. 1993 Framing: toward clarification of a fractured paradigm. J. Commun. **43**, 51–58.

[35] Kueffer C, Larson BMH. 2014 Responsible use of language in scientific writing and science communication. BioScience **64**, 719–724. (10.1093/biosci/biu084)

[36] Fish RD, Austen GE, Bentley JW, Dallimer M, Fisher JC, Irvine KN, Bentley PR, Nawrath M, Davies ZG. 2024 Language matters for biodiversity. BioScience **74**, 333–339. (10.1093/biosci/biae014) 38854634 PMC11153204

[37] Walker JMM, Godley BJ, Nuno A. 2019 Media framing of the Cayman turtle farm: implications for conservation conflicts. J. Nat. Conserv. **48**, 61–70. (10.1016/j.jnc.2019.01.001)

[38] Hammond NL, Dickman A, Biggs D. 2022 Examining attention given to threats to elephant conservation on social media. Conservat. Sci. and Prac. **4**, e12785. (10.1111/csp2.12785)

[39] Iyengar S. 1990 Framing responsibility for political issues: the case of poverty. Polit. Behav. **12**, 19–40. (10.1007/BF00992330)

[40] Levin IP, Schneider SL, Gaeth GJ. 1998 All frames are not created equal: a typology and critical analysis of framing effects. Organ. Behav. Hum. Decis. Process. **76**, 149–188. (10.1006/obhd.1998.2804) 9831520

[41] Tversky A, Kahneman D. 1981 The framing of decisions and the psychology of choice. Science **211**, 453–458. (10.1126/science.7455683) 7455683

[42] Gross K. 2008 Framing persuasive appeals: episodic and thematic framing, emotional response, and policy opinion. Polit. Psychol. **29**, 169–192. (10.1111/j.1467-9221.2008.00622.x)

[43] Han G, Wang X. 2012 Understanding ‘Made in China’ valence framing and product-country image. J. Mass Commun. Q **89**, 225–243. (10.1177/1077699012439)

[44] Bielby J, McMillan KM, Davies G. 2025 It’s not fur: newspaper article reporting of abandonment and relinquishment of pets exhibit taxonomic biases in framing and language use. Dryad digital repository (10.5061/dryad.59zw3r2kj) PMC12308231 40740710

[45] Landis JR, Koch GG. 1977 The measurement of observer agreement for categorical data. Biometrics **33**, 159–174. 843571

[46] R Core Team. 2022 R: a language and environment for statistical computing. Vienna, Austria: R Foundation for Statistical Computing. See https://www.R-project.org/.

[47] Wickham H *et al*. 2019 Welcome to the Tidyverse. J. Open Source Softw. **4**, 1686. (10.21105/joss.01686)

[48] Wickham H, François R, Henry L, Müller K, Vaughan D. 2023 dplyr: A Grammar of Data Manipulation. R package version 1.1.4, https://github.com/tidyverse/dplyr. See https://dplyr.tidyverse.org.

[49] Wickham H. 2016 ggplot2: elegant graphics for data analysis. See https://ggplot2.tidyverse.org.

[50] Agresti A. 2012 Categorical data analysis. vol. 792. Hoboken, NJ: John Wiley & Sons.

[51] Bradley MM, Lang PJ. 1999 Affective norms for English words (ANEW): instruction manual and affective ratings. Technical report C-1, The Center for Research in Psychophysiology, University of Florida.

[52] Mohammad SM, Turney PD. 2013 Crowdsourcing a word–emotion association lexicon. Comput. Intell. **29**, 436–465. (10.1111/j.1467-8640.2012.00460.x)

[53] Manning CD, Raghavan P, Schütze H. 2008 Introduction to information retrieval, pp. 19–48. Cambridge, UK: Cambridge University Press.

[54] Silge J, Robinson D. 2016 tidytext: text mining and analysis using tidy data principles in R. J. Open Source Soft. **1**, 37. (10.21105/joss.00037)

[55] Wickham H. 2022 _stringr: simple, consistent wrappers for common string operations. R package version 1.5.0. See https://CRAN.R-project.org/package=stringr.

[56] Bouchet-Valat M. 2023 SnowballC: snowball stemmers based on the C ‘libstemmer’ UTF-8 library. R package version 0.7.1. See https://CRAN.R-project.org/package=SnowballC.

[57] Bielby J, Austen G, McMillan K, Wafflart S. 2025. Data from: Exploring Media Representation of the Exotic Pet Trade, with a Focus on Welfare: Taxonomic, Framing, and Language Biases in Peer-Reviewed Publications and Newspaper Articles. Dryad (10.5061/dryad.m0cfxppf6) PMC11880843 40046661

[58] Gray PB, Young SM. 2011 Human–pet dynamics in cross-cultural perspective. Anthrozoös **24**, 17–30. (10.2752/175303711x12923300467285)

[59] Alves RRN, Rocha LA. 2018 Fauna at home: animals as pets. In Ethnozoology (eds RRN Alves, UP Albuquerque), pp. 303–321. Cambridge, MA: Academic Press. (10.1016/B978-0-12-809913-1.00016-8)

[60] Wensley S, Betton V, Gosschalk K, Hooker R, Main DCJ, Martin N, Tipton E. 2021 Driving evidence‐based improvements for the UK’s ‘Stressed. Lonely. Overweight. Bored. Aggressive. Misunderstood … but loved’ companion animals. Vet. Rec. **189**, e7. (10.1002/vetr.7) 33818804

[61] Chua D, Rand J, Morton J. 2017 Surrendered and stray dogs in Australia—estimation of numbers entering municipal pounds, shelters and rescue groups and their outcomes. Animals **7**, 50. (10.3390/ani7070050) 28704949 PMC5532565

[62] Fatjó J, Bowen J, García E, Calvo P, Rueda S, Amblás S, Lalanza JF. 2015 Epidemiology of dog and cat abandonment in Spain (2008–2013). Animals **5**, 426–441. (10.3390/ani5020364) 26479243 PMC4494419

[63] Protopopova A, Gunter LM. 2017 Adoption and relinquishment interventions at the animal shelter: a review. Anim. Welf. **26**, 35–48. (10.7120/09627286.26.1.035)

[64] Stavisky J, Brennan ML, Downes M, Dean R. 2012 Demographics and economic burden of un-owned cats and dogs in the UK: results of a 2010 census. BMC Vet. Res. **8**, 1–10. (10.1186/1746-6148-8-163) 22974242 PMC3514250

[65] Harrup AJ, Rooney N. 2020 Current welfare state of pet guinea pigs in the UK. Vet. Rec. **186**, 282–282. (10.1136/vr.105632) 32054719

[66] Arahori M, Kuroshima H, Hori Y, Takagi S, Chijiiwa H, Fujita K. 2017 Owners’ view of their pets’ emotions, intellect, and mutual relationship: cats and dogs compared. Behav. Process. **141**, 316–321. (10.1016/j.beproc.2017.02.007) 28267573

[67] Greenebaum J. 2004 It’s a dog’s life: elevating status from pet to ‘fur baby’ at yappy hour. Soc. Anim. **12**, 17–135. (10.1163/1568530041446544)

[68] Haddon C, Burman OHP, Assheton P, Wilkinson A. 2021 Love in cold blood: are reptile owners emotionally attached to their pets? Anthrozoös **34**, 739–749. (10.1080/08927936.2021.1926711)

[69] Nagabaskaran G, Burman OHP, Hoehfurtner T, Wilkinson A. 2021 Environmental enrichment impacts discrimination between familiar and unfamiliar human odours in snakes (Pantherophis guttata). Appl. Anim. Behav. Sci. **237**, 105278. (10.1016/j.applanim.2021.105278)

[70] Santacà M, Miletto Petrazzini ME, Agrillo C, Wilkinson A. 2019 Can reptiles perceive visual illusions? Delboeuf illusion in red-footed tortoise (Chelonoidis carbonaria) and bearded dragon (Pogona vitticeps). J. Comp. Psychol. **133**, 419–427. (10.1037/com0000176) 30896231

[71] Freiburger T, Miller N, Skinner M. 2024 Olfactory self-recognition in two species of snake. Proc. R. Soc. B **291**, 20240125. (10.1098/rspb.2024.0125) PMC10987230 38565155

[72] Pasmans F, Bogaerts S, Braeckman J, Cunningham AA, Hellebuyck T, Griffiths RA, Sparreboom M, Schmidt BR, Martel A. 2017 Future of keeping pet reptiles and amphibians: towards integrating animal welfare, human health and environmental sustainability. Vet. Rec. **181**, 450–450. (10.1136/vr.104296) 29051315

[73] Crisante A, Burman OHP, Wilkinson A. 2023 Does ownership impact perception of reptile cognitive abilities and welfare needs? Appl. Anim. Behav. Sci. **268**, 106067. (10.1016/j.applanim.2023.106067)

[74] Nilsen AB, Fylkesnes S, Mausethagen S. 2017 The linguistics in othering: teacher educators’ talk about cultural diversity. Reconceptualizing Educ. Res. Methodol **8**, 40–50. (10.7577/rerm.2556)

[75] Chen Z, Jin J, Li M. 2022 Does media coverage influence firm green innovation? The moderating role of regional environment. Technol. Soc. **70**, 102006. (10.1016/j.techsoc.2022.102006)

[76] Ijioma NE, Nze C. 2022 Evaluating the influence of social media use in COVID-19 vaccine hesitancy of residents of Owerri metropolis. Adv. Journal. Commun. **10**, 10–24. (10.4236/ajc.2022.101002)

[77] Hunter LY. 2023 Social media, disinformation, and democracy: how different types of social media usage affect democracy cross-nationally. Democratization **30**, 1040–1072. (10.1080/13510347.2023.2208355)

[78] Wu CST, Wong RSM, Chu WH. 2018 The association of pet ownership and attachment with perceived stress among Chinese adults. Anthrozoös **31**, 577–586. (10.1080/08927936.2018.1505269)

[79] Joseph N, Chandramohan AK, D’souza AL, Hariram S, Nayak AH. 2019 Assessment of pet attachment and its relationship with stress and social support among residents in Mangalore city of south India. J. Vet. Behav. **34**, 1–6. (10.1016/j.jveb.2019.06.009)

[80] Weldon AV, Campera M, Zhang X, Ni Q, Zhu WW, Nijman V, Nekaris KAI. 2021 Perceptions of animal welfare and exotic pet ownership in China. Anim. Welf. **30**, 169–178. (10.7120/09627286.30.2.169)

[81] Guo X, Meijboom FL. 2023 The development of animal welfare science in China: an explorative analysis. Anim. Welf. **32**, e72. (10.1017/awf.2023.93) 38487417 PMC10936407

[82] Phillips CJC *et al*. 2012 Students’ attitudes to animal welfare and rights in Europe and Asia. Anim. Welf. **21**, 87–100. (10.7120/096272812799129466)

[83] Rowan AN, Kartal T, Hadidian J. 2019 Cat demographics & impact on wildlife in the USA, the UK, Australia and New Zealand: facts and values. J. Appl. Anim. Ethics Res. **2**, 7–37. (10.1163/25889567-12340013)

[84] Maher J, Wyatt T. 2021 European illegal puppy trade and organised crime. Trends Organ. Crime **24**, 506–525. (10.1007/s12117-021-09429-8) 34456550 PMC8382934

[85] Pet Food Manufacturers Association and Federation of British Herpetologists. 2021 Reptile and amphibian keepers’ survey. See https://www.thefbh.org/publications (accessed 8 May 2024).

[86] Bielby J, Ferguson A, Rendle M, McMillan KM. 2023 Online classified adverts reflect the broader United Kingdom trade in turtles and tortoises rather than drive it. PLoS One **18**, e0288725. (10.1371/journal.pone.0288725) 37440593 PMC10343072

[87] Hoehfurtner T, Wilkinson A, Walker M, Burman OHP. 2021 Does enclosure size influence the behaviour & welfare of captive snakes (Pantherophis guttatus)? Appl. Anim. Behav. Sci. **243**, 105435. (10.1016/j.applanim.2021.105435)

[88] Hollandt T, Baur M, Wöhr AC. 2021 Animal-appropriate housing of ball pythons (Python regius)—behavior-based evaluation of two types of housing systems. PLoS One **16**, e0247082. (10.1371/journal.pone.0247082) 34043634 PMC8158952

[89] Moszuti SA, Wilkinson A, Burman OHP. 2017 Response to novelty as an indicator of reptile welfare. Appl. Anim. Behav. Sci. **193**, 98–103. (10.1016/j.applanim.2017.03.018)

